# The predictive value of lncRNA MIR31HG expression on clinical outcomes in patients with solid malignant tumors

**DOI:** 10.1186/s12935-020-01194-y

**Published:** 2020-04-07

**Authors:** Chao Tu, Xiaolei Ren, Jieyu He, Shuangqing Li, Lin Qi, Zhixi Duan, Wanchun Wang, Zhihong Li

**Affiliations:** 1 Department of Orthopedics, The Second Xiangya Hospital, Central South University, No 139 Middle Renmin Road, Changsha, Hunan 410011 China; 2 Hunan Key Laboratory of Tumor Models and Individualized Medicine, The Second Xiangya Hospital, Central South University, Changsha, Hunan 410011 China; 3 Department of Geriatrics, The Second Xiangya Hospital, Central South University, Changsha, Hunan 410011 China

**Keywords:** LncRNA MIR31HG,, Sarcoma, Tumor, Prognosis, Overall survival

## Abstract

**Background:**

Emerging studies have explored the prognostic value of MIR31HG in cancers, but its role remains elusive. Herein, we aimed to summarize the prognostic potential of MIR31HG in this study.

**Methods:**

Several databases were searched for literature retrieval on Dec 5, 2019. Overall and subgroup analyses were conducted to measure the relationship between MIR31HG expression and clinical outcomes. Moreover, GEPIA was applied for validation of prognostic value of MIR31HG in tumor patients in TCGA dataset.

**Results:**

Overall, seventeen studies with 2573 patients were enrolled. Compared to counterparts, those patients with high MIR31HG expression tended to have shorter RFS. Notably, MIR31HG overexpression predicted unfavorable OS in lung cancer. By contrast, gastrointestinal cancer patients with elevated MIR31HG expression predicted better OS and disease-free survival. Additionally, MIR31HG overexpression was significantly associated with worse clinicopathological features including advanced tumor stage and LNM in lung cancer, but favorable clinical characteristics in gastrointestinal cancer. Moreover, the positive association between MIR31HG and OS in lung cancer was further confirmed in TCGA dataset.

**Conclusion:**

Overexpression of MIR31HG suggested remarkable association with poor prognosis in terms of OS, tumor stage, and LNM in lung cancer, but favorable prognosis in gastrointestinal cancer. Therefore, MIR31HG may serve as a promising prognostic biomarker in multiple cancers.

## Background

Cancer is the major cause of death and public health problem worldwide [1]. In spite of notable continuous advances in cancer treatment over the last decades, the patients’ survival rate remains disappointed [2]. Nowadays, only a minority of patients in early clinical stage could benefit from curative approaches, while the rest patients, particularly those with advanced stage, may be resistant to conventional treatments [3]. Against this backdrop, it is necessary to explore reliable biomarkers harboring sufficient sensitivity and specificity for early diagnosis and attractive targets for therapeutic intervention before reaching to advanced stages [4, 5].

Long non-coding RNAs (lncRNAs), longer than 200 nucleotides (nt), are defined as highly conserved non-coding RNAs with limited or no protein-coding capacity [6]. LncRNA are involved in diverse physiological and pathological cellular processes [7] by promoting or restraining the protein-coding genes expression [8]. Accumulating evidences have showed that aberrant expression of lncRNA could drive tumor phenotypes, including initiation, invasion, and metastasis, via interacting with other cellular macromolecules [9]. More recently, emerging lncRNAs have been identified and characterized as crucial factors in tumorigenesis, with certain reports highlighting them as biomarkers and targets in various cancers, such as breast cancer anti-estrogen resistance 4 (BCAR4) [10], small nucleolar RNA host gene 16 (SNHG16) [11], and TTN antisense RNA 1 (TTN-AS1) [12].

LncRNA microRNA-31 host gene (MIR31HG), also known as *LOC554202* [13], is a newly identified lncRNA with a length of 2166 nt [2, 4]. Meanwhile, MIR31HG was also named as LncHIFCAR (long noncoding HIF-1α co-activating RNA) since it is a hypoxia-inducible lncRNA [4]. P16 (INK4A) is a well-known tumor suppressor, MIR31HG was reported to negatively regulate p16-dependent senescence phenotype [14], suggesting a potential role in carcinogenesis.

Recently, an increasing number of studies have been exploring the prognostic association between MIR31HG and cancers. Abnormal expression of MIR31HG has been reported in a diverse range of cancer types, including osteosarcoma [15], chordoma [16], colorectal cancer (CRC) [17–19], hepatocellular carcinoma (HCC) [6], lung cancer [2, 20–24], gastric cancer [25], bladder cancer [26], pancreatic ductal adenocarcinoma (PDAC) [7], oral squamous cell carcinoma (OSCC) [4], breast cancer [13, 27], cervical cancer [28], vulvar squamous cell carcinoma (VSCC) [29], and esophageal squamous cell carcinoma (ESCC) [30, 31]. Furthermore, MIR31HG expression was remarkably associated with survival time, tumor-node metastasis (TNM) stage and carcinogenesis of certain cancers. However, the prognostic significance of MIR31HG may be jeopardized by controversial results, ethnic or geographical limitations, and limited sample size. For example, Sun et al. [30] reported that MIR31HG expression was significantly elevated in ESCC tissues and plasma, and MIR31HG could promote ESCC cell proliferation, migration and invasion [30]. On the contrary, another study revealed that MIR31HG appeared to have lower expression in ESCC tissues and patients with reduced MIR31HG were correlated with malignant clinical features. The contradictory expression patterns of MIR31HG were also found in breast cancer [13, 27], CRC [19, 32, 33] and gastric cancer [25, 34]. In view of these conflicting data, for the first time, we performed this comprehensive meta-analysis of the current evidences in order to determine the predictive value of MIR31HG in various carcinomas.

## Materials and methods

### Literature search strategy

Studies in relevant with MIR31HG and cancer prognosis were thoroughly searched via PubMed, Web of Science, and Cochrane library (up to Dec 5, 2019). The search strategy was used as following: (“Cancer” OR “Malignancy” OR “Sarcoma” OR “Tumor” OR “Neoplasm” OR “Carcinoma”) AND (“MIR31HG” OR “*LOC554202*″ OR “LncHIFCAR”) AND (“Long non-coding RNA” OR “LncRNA” OR “long untranslated RNA”OR “lincRNA”) AND (“Prognosis” OR “Prognostic”). The reference lists of enrolled studies were further reviewed to identify relevant articles.

### Study selection criteria and data extraction

Studies were included based on the following inclusion criteria: [1] original studies; [2] the diagnosis of cancer was validated by pathology and histology; [3] studies exploring the correlation between MIR31HG and cancers; [4] MIR31HG expression levels were measured and divided into high or low expression group; [5] reporting of at least one of the endpoints: overall survival (OS), disease-free survival (DFS) and relapse-free survival (RFS), et al.; [6] sufficient data: the hazard ratios (HRs) or odds ratios (ORs) with 95% confidence intervals (CIs) or K-M survival curves were shown; [7] the more recent article was selected if duplicated researches were reported.

On contrary, studies were excluded due to following exclusion criteria: [1] article type: meta-analysis, reviews, editorial material, or case reports; [2] in vivo or in vitro studies without clinical data; [3] sufficient survival data were not provided or cannot be extracted; [4] full text was not accessible; [5] duplicates.

Two independent investigators (CT and XLR) screened and extracted the data from selected studies according to the criteria abovementioned. Any divergence views were resolved by another senior researcher (WCW) to reach a consensus. The following items were provided: first author, year of publication, country, sample size, tumor type, detection methods, follow-up time, and prognostic outcomes. HRs (ORs) and 95% CIs were obtained from the original texts in eligible studies as provided. If not, Engauge Digitizer (version 4.1) would be used to calculate the HR through K-M survival curve as previously described [11].

### Quality assessment

Two investigators (JYH and SQL) assessed the quality of studies independently in accordance with the Newcastle–Ottawa scale (NOS). Usually, studies with NOS ≥ 6 were considered as high-quality. The scores of all eligible studies in this study ranged from 6 to 8, which indicated high quality.

### Prognosis analysis in GEPIA using TCGA dataset

Gene Expression Profiling Interactive Analysis (GEPIA) (http://gepia.cancer-pku.cn/index.html) based on The Cancer Genome Atlas (TCGA) data [35] was further searched for cross-validation of the expression pattern and prognostic significance of MIR31HG in cancers.

### Publication bias and sensitivity analysis

Sensitivity analysis was performed by omitting individual study consecutively to evaluate their impact on this study. Begg’s test and Egger’s test were both utilized to evaluate the publication bias.

### Statistical analysis

Data analysis was conducted by using STATA (version 12.0) and RevMan (Version 5.3). A two-tailed P value less than 0.05 was considered statistically significant. HRs and corresponding 95% CIs were adopted to evaluate the relation between MIR31HG expression and cancer prognosis as well as other clinicopathological characteristics. Heterogeneity of the included studies was assessed by Cochrane’s Q statistic and *I*^*2*^ test. If p<0.05, or *I*^*2*^≥ 50%, a random-effects model shall be applied to calculate the pooled results due to substantial heterogeneity. Otherwise, a fixed-effects model would be used. Subgroup analysis was performed as per the tumor type and follow-up months in case of obvious heterogeneity among the selected studies.

## Results

### Characteristics of the included studies

**Fig. 1:**
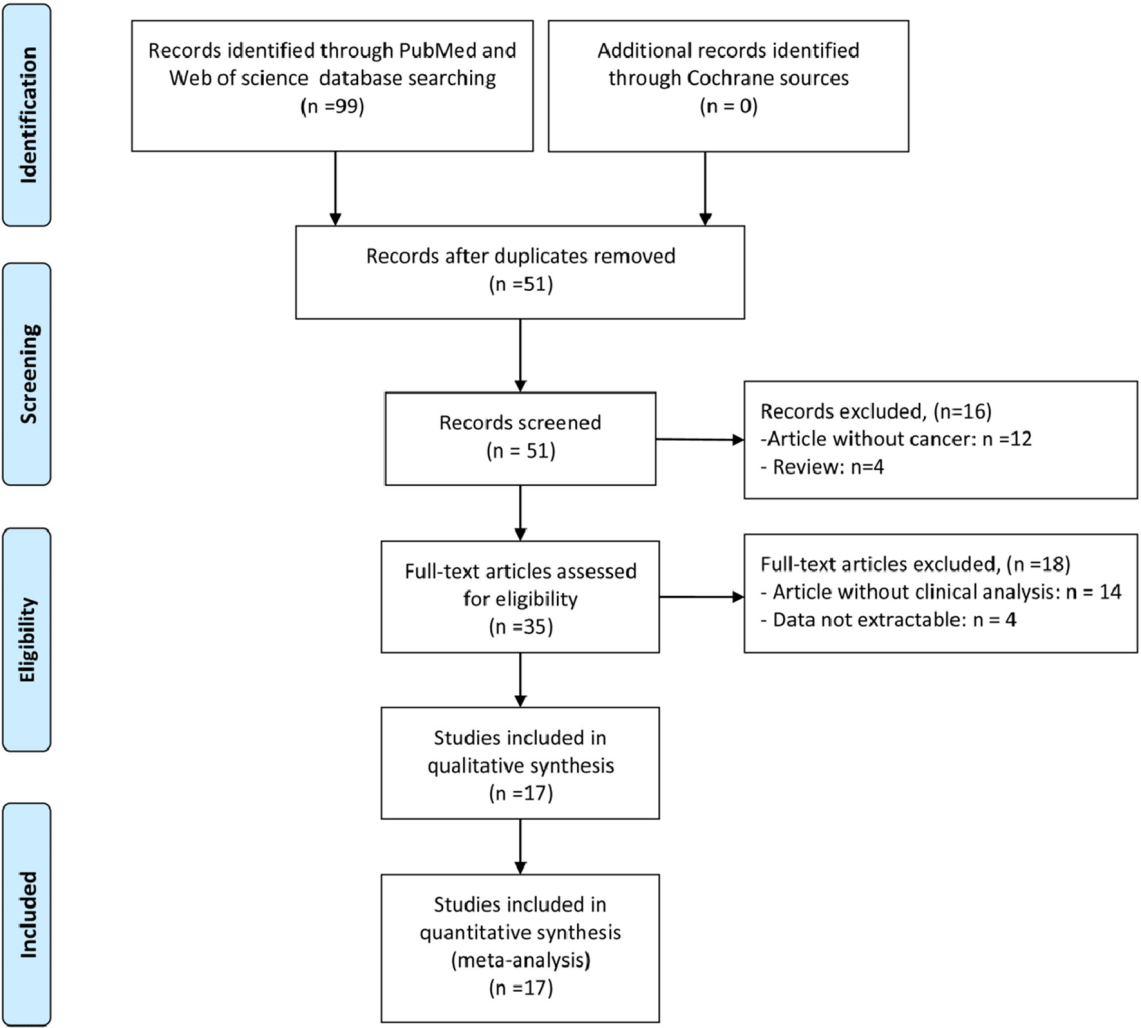
The PRISMA flow diagram of study selection

According to the screening criteria, seventeen articles comprising 2573 patients were included in our study [1, 2, 4, 6, 15, 17, 19, 23, 25, 26, 28–33, 36]. All the included studies were related to lncRNA MIR31HG in cancer and comprised clinical data. The study screening flow diagram was shown in Fig. 1. Among the included seventeen articles, twelve types of cancers were identified, including osteosarcoma, cervical cancer, gastric cancer, bladder cancer, CRC, lung adenocarcinoma (LUAD), non-small-cell lung carcinoma (NSCLC), HCC, VSCC, ESCC, OSCC, and laryngeal squamous cell carcinoma (LSCC). The included studies were performed between 2015 and 2019, and almost all of them were originated in China except one from Norway [17]. The sample size ranged from 16 to 1265. Survival analysis regarding OS, DFS and RFS were measured in twelve studies with follow-up time ranging from 25 to 100 months. Quantitative reverse transcription polymerase chain reaction (qRT-PCR) was utilized for analysis of the relative expression level of MIR31HG in tumor tissues except one used RNA sequencing [4]. The cut-off values of MIR31HG expression were median value. NOS scores were calculated for the quality evaluation with value ranging from 6 to 8, indicating that the enrolled studies were of high quality. Details of the abovementioned characteristics of the studies were presented in Table 1.

**Table 1:** Baseline characteristics of eligible studies

Authors	Publication year	Country	Cancer type	Sample size	Follow-up months	Detection method	Clinical outcomes	Analysis method	NOS
Chen et al. [27]	2017	China	Cervical cancer	120	60	qRT-PCR	OS	Multivariate	7
Ding et al. [18]	2015	China	CRC	48	–	qRT-PCR	–	Multivariate	6
Eideet al. [16]	2019	Norway	CRC	1265	60	qRT-PCR	RFS	Multivariate	7
He et al. [25]	2016	China	Bladder cancer	55	–	qRT-PCR	–	Multivariate	6
Li et al. [31]	2018	China	CRC	157	100	qRT-PCR	OS, DFS	Multivariate	7
Ni et al. [28]	2016	China	VSCC	16	–	qRT-PCR	–	Multivariate	6
Nie et al. [24]	2016	China	Gastric cancer	42	40	qRT-PCR	OS	Multivariate	6
Qin et al. [22]	2018	China	LUAD	112	85	qRT-PCR	OS	Multivariate	7
Ren , et al. [30]	2017	China	ESCC	185	60	qRT-PCR	OS	Multivariate	6
Shih et al. [4]	2017	China	OSCC	42	40	RNA-seq	OS, RFS	Multivariate	7
Sun et al. [29]	2018	China	ESCC	53	–	qRT-PCR	–	Multivariate	6
Sun et al. [14]	2019	China	Osteosarcoma	40	–	qRT-PCR	–	Multivariate	6
Wang et al. [35]	2018	China	LSCC	60	85	qRT-PCR	OS, RFS	Multivariate	7
Wu et al. [40]	2019	China	NSCLC	50	85	qRT-PCR	OS	Multivariate	8
Yan et al. [5]	2018	China	HCC	42	25	qRT-PCR	OS	Multivariate	7
Yang et al. [32]	2016	China	CRC	178	70	qRT-PCR	OS, DFS	Multivariate	7
Zheng et al. [2]	2019	China	NSCLC	88	50	qRT-PCR	OS	Multivariate	8

*CRC* colorectal cancer, *DFS* disease-free survival, *ESCC* esophageal squamous cell carcinoma, HCC hepatocellular carcinoma, LSCC laryngeal squamous cell cancer, *LUAD* lung adenocarcinoma, *NOS* Newcastle-Ottawa Scale, *NSCLC* Non–small cell lung cancer, *OS* overall survival, *OSCC* oral squamous cell carcinoma, *qRT-PCR* real-time quantitative reverse transcription polymerase chain reaction, *RFS* relapse-free survival, VSCC vulvar squamous cell carcinoma

### Expression of lncRNA MIR31HG as a prognosis marker in various cancers

**Fig. 2:**
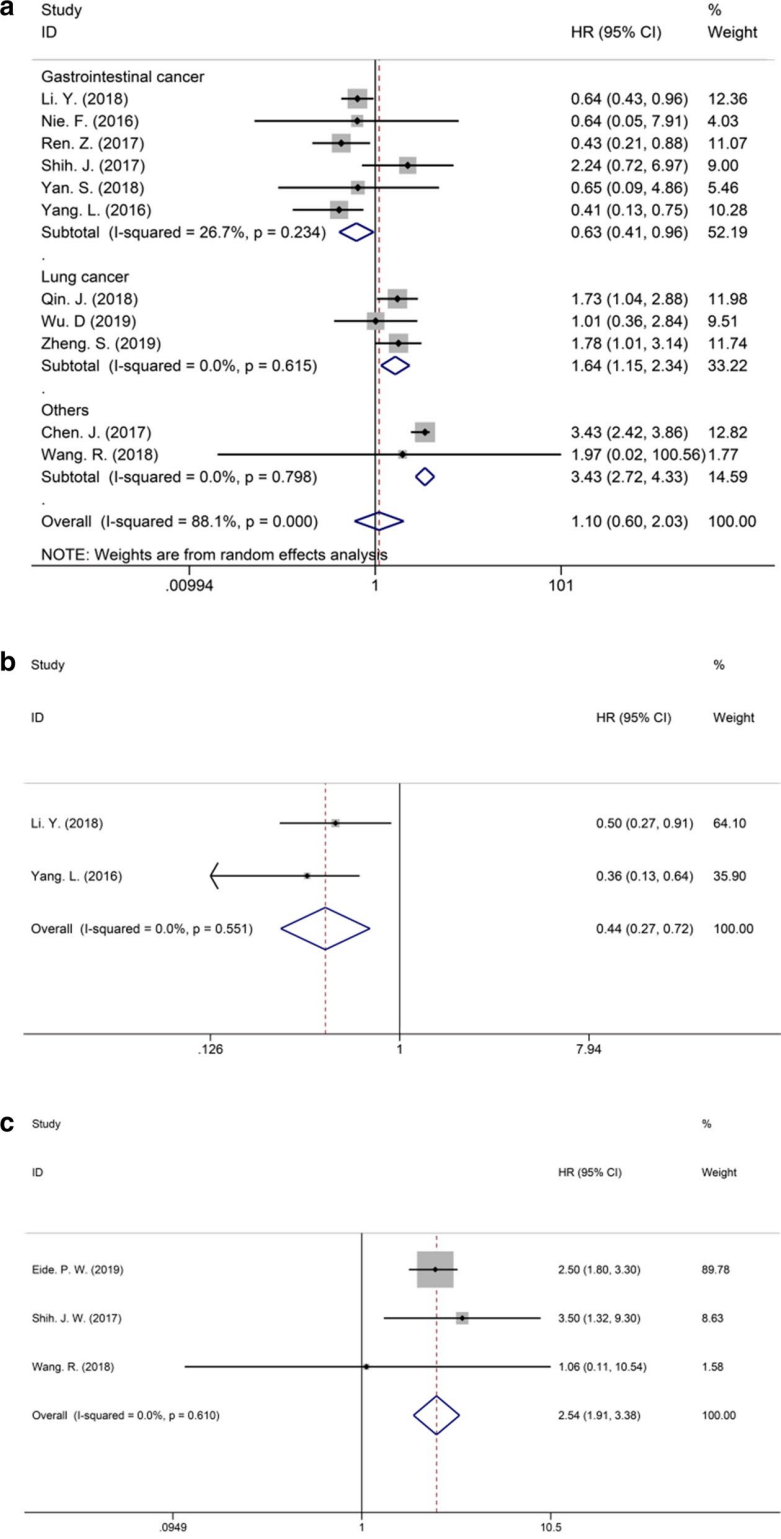
Forest plots describing the overall and subgroup analysis of HRs for association between MIR31HG expression and OS (**a**), DFS (**b**), and RFS (**c**). *CI* confidence interval, *DFS* disease-free survival, *HR* hazard ratio, *OS* overall survival, *RFS* relapse-free survival

In this study, the association between prognosis and MIR31HG expression were reported in twelve studies. Eleven studies were included for OS meta-analysis. As the result of meta-analysis exhibited obvious heterogeneity (P < 0.001, I2 = 88.1%), subgroup analysis was further conducted to calculate the pooled HRs or ORs and 95% CIs in which the patients were stratified by cancer type (gastrointestinal cancer, lung cancer or others), or follow-up time (more or less than 50 months), as shown in Fig. 2a and Additional file 1: Figure S1, respectively. The gastrointestinal cancer included ESCC, gastric cancer, HCC, and CRC, while the lung cancer consisted of NSCLC and LUAD. Besides, OSCC, LSCC and cervical cancer were grouped in other cancers. The random-effect model was applied to reduce the possible bias. All the pooled HRs and 95% CIs of the overall and subgroup analysis were shown in Table 2. The results demonstrated that elevated expression of the MIR31HG predicted favorable OS in gastrointestinal cancer (HR = 0.56, 95% CI 0.410.77), but unfavorable OS in lung cancer (HR = 1.64, 95% CI 1.152.34) and other cancers (HR = 3.37, 95% CI 2.694.23) (Fig. 2a and Table 2). Interestingly, the pooled results also showed that overexpression of MIR31HG predicted shorter OS in studies with follow-up months less than 50, while no correlation was found in studies with follow-up more than 50 months (Figure S1A).

**Table 2:** The pooled results of association between clinical prognosis and MIR31HG expression

Outcomes	Studies	Participants	HRs and 95% CIs	Model	Heterogeneity
					P, *I*^2^
OS					
Total	11	1096	1.10 (0.60, 2.03)	Random	< 0.001, 88.1%
Gastrointestinal cancer	5	604	0.56 (0.41, 0.77)	Random	0.825, 0%
Lung cancer	3	270	1.64 (1.15, 2.34)	Random	0.615, 0%
Other cancers	3	222	3.37 (2.69, 4.23)	Random	0.746, 0%
DFS	2	335	0.44 (0.27, 0.72)	Fixed	0.551, 0%
RFS	3	1367	2.54 (1.91, 3.38)	Fixed	0.610, 0%

*CI* confidence interval, *DFS* disease-free survival, *HR* hazard ratio, *OS* overall survival, *RFS* relapse-free survival

Moreover, two studies on CRC for DFS and three studies on various cancers reported available data for RFS were further pooled for measurement. The HR and 95% CI (HR = 0.44, 95% CI 0.270.72) indicated overexpression of MIR31HG predicted favorable DFS in CRC (Fig. 2b). However, overexpression of MIR31HG was correlated with poor RFS (HR = 2.54, 95% CI 1.913.38) (Fig. 2c).

### Association between MIR31HG expression and other clinicopathologic parameters

**Fig. 3:**
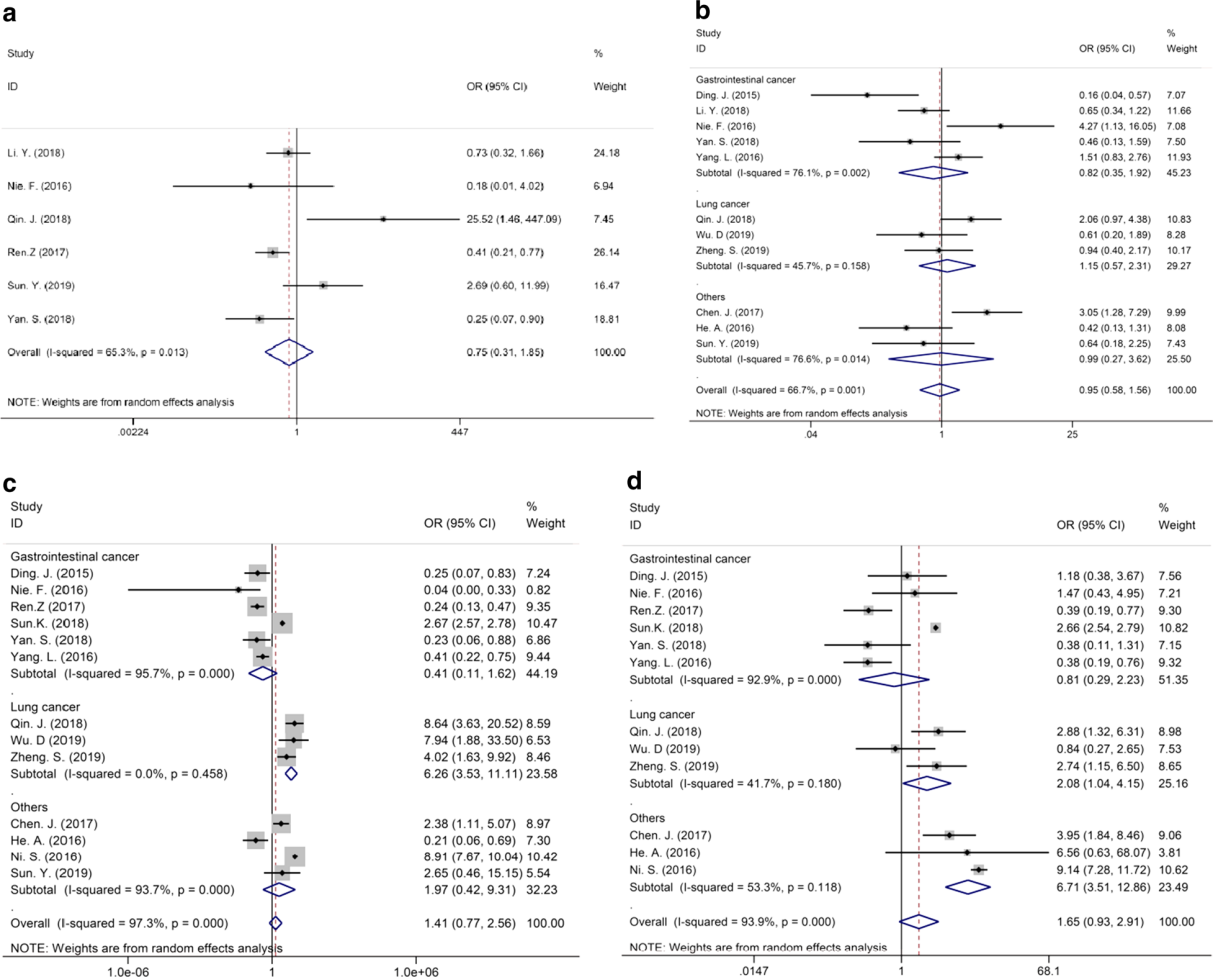
Forest plot describing overall and stratified analysis of relationship between MIR31HG expression and clinicopathological parameters, including DM (**a**), tumor size (**b**), advanced clinical stage (**c**), and LNM (**d**). *DM* distant metastasis, *LNM* lymph node metastasis

The association between MIR31HG and clinicopathologic features, including clinical stage, lymph node metastasis (LNM), distant metastasis (DM), and tumor size were further analyzed by ORs and their 95% CIs. There was no significant difference in MIR31HG expression detected in DM (OR = 0.75, 95% CI 0.311.85) and tumor size (OR = 0.95, 95% CI 0.581.56), even if the stratified analysis were performed by cancer type (Fig. 3a, b). Although, the pooled overall OR and 95% CI showed no significant difference in MIR31HG expression analysis in various clinical stage (OR = 1.41, 95% CI 0.772.56) and LNM (OR = 1.65, 95% CI 0.932.91), after subgroups analysis by cancer type, the results suggested that upregulated expression of MIR31HG indicated the advanced clinical stage (OR = 6.26, 95% CI 3.5311.11) and higher risk of LNM in lung cancer (OR = 2.08, 95% CI 1.044.15) (Fig. 3c, d).

### Sensitivity analysis

**Fig. 4:**
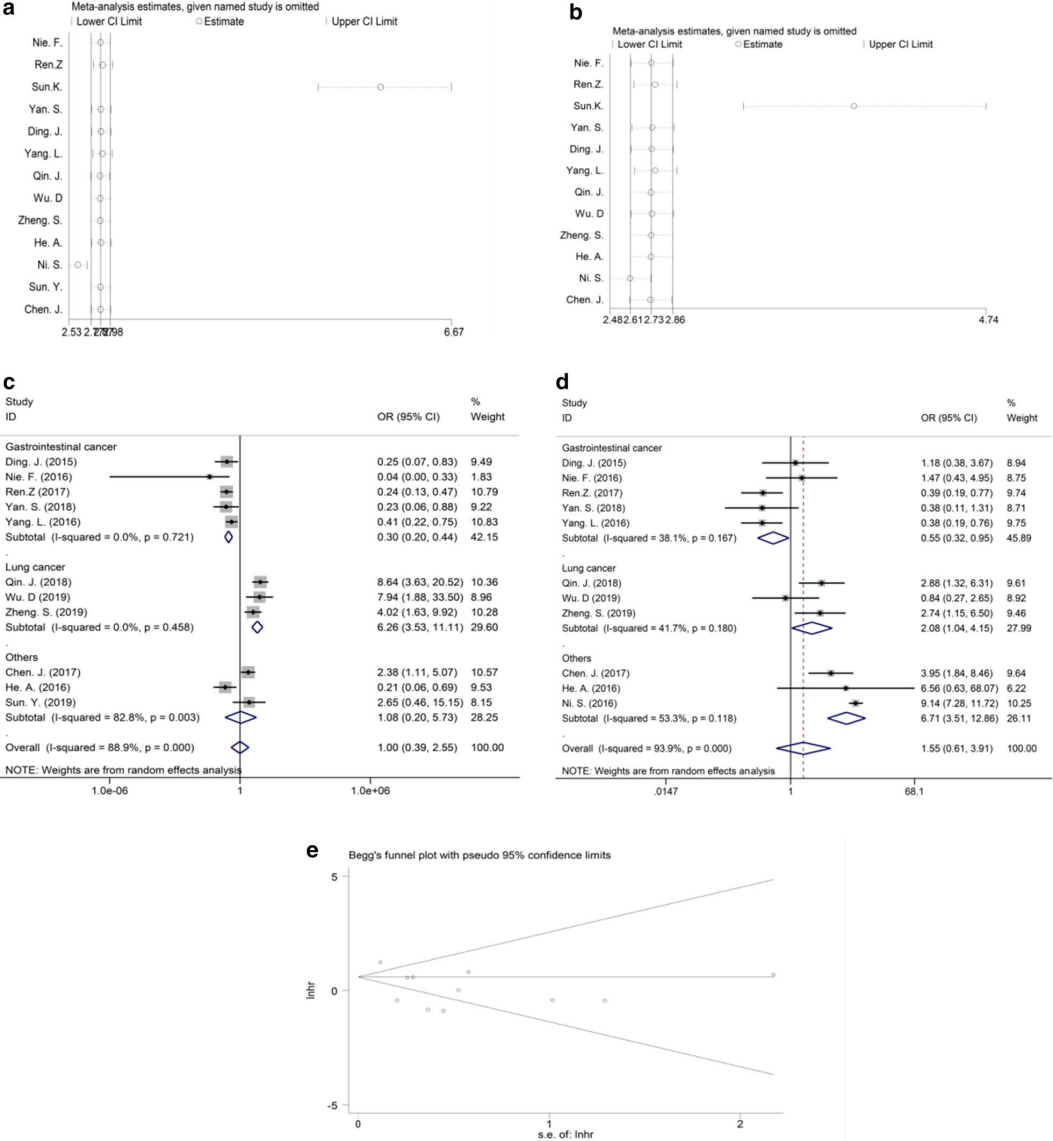
Sensitivity analysis of each study reporting clinical stages (**a**) and LNM (**b**). Overall and subgroup analysis of association between MIR31HG expression and advanced clinical stage (**c**), or LNM (**d**) after deletion according to the sensitive analysis. Begg’s funnel plot for included studies reporting OS (**e**) *LNM* lymph node metastasis, *OS* overall survival

Sensitivity analysis was performed to the studies which had more than two articles. All results showed that this meta-analysis was stable except the analysis of clinical stage (Fig. 4a) and LNM (Fig. 4b). After dropping the research performed by Sun et al., the pooled ORs and 95% CIs showed that elevated expression of MIR31HG were negatively associated with clinical stage (OR = 0.30, 95% CI 0.200.44) and LNM risk in gastrointestinal cancer (OR = 0.55, 95% CI 0.320.95) (Fig. 4c, d).

### Publication bias

Publication bias was measured for studies reporting OS by using the funnel plot, Begg’s and Egger’s test. No significant publication bias was observed as the funnel plot showed symmetry, as demonstrated in Fig. 4e. Moreover, the *P* values for Begg’s and Egger’s test were 0.533 and 0.111, respectively, also indicating the absence of publication bias.

### Prognostic analysis in GEPIA using TCGA and GTEx dataset

**Fig. 5:**
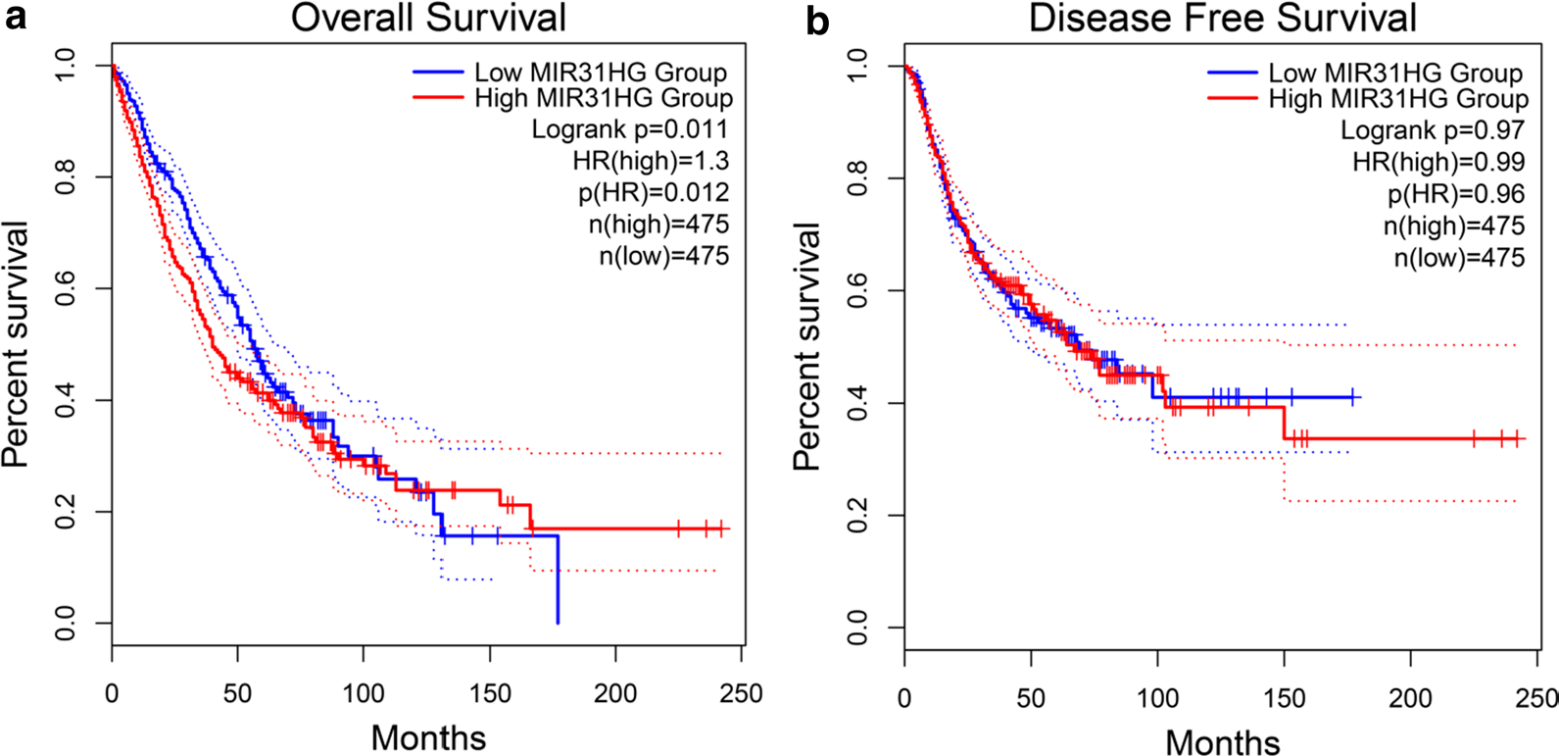
The prognostic value of MIR31HG in lung cancer patients with tumors in TCGA dataset, including OS (**a**), DFS (**b**). *DFS* disease-free survival, *HR* hazard ratio, *OS* overall survival, *TCGA* The Cancer Genome Atlas

Furthermore, the relationships between expression of MIR31HG and OS/DFS in lung cancers were validated in TCGA and GTEx dataset by using GEPIA as previously described [11]. The results indicated that high MIR31HG expression was correlated with significant shorter OS (HR = 1.3, *P *= 0.012), but not DFS (HR = 0.99, *P *= 0.96) (Fig. 5). Besides, the aberrant expression pattern of MIR31HG in multiples cancers and matched normal tissues were also identified, including sarcoma (SARC), LUAD, lung squamous cell carcinoma (LUSC), cervical squamous cell carcinoma and endocervical adenocarcinoma (CESC), pancreatic adenocarcinoma (PAAD), bladder urothelial carcinoma (BLCA), breast invasive carcinoma (BRCA), colon adenocarcinoma (COAD), rectum adenocarcinoma (READ), stomach adenocarcinoma (STAD), and esophageal carcinoma (ESCA) (Additional file 1: Figure S1b, c).

## Discussion

Identifying correlative biomarkers with high sensitivity and specificity is of great importance in cancer diagnosis and treatment [5]. Recently, with the development of next generation sequencing, lncRNAs are revealed as multifaceted regulators in a wide range of cellular homeostasis [37] via participating in epigenetic, transcriptional and posttranscriptional regulation of genes [26]. Increasing evidences have established a strong relationship between dysfunction of lncRNAs and cell fate determination as well as disease pathogenesis, such as aging [38], arthritis [39], and cancer [13]. LncRNA MIR31HG, locating at human chromosome 9p21.3 [40] with transcription regulated by methylation of the promoter region [27], was found aberrantly expressed in several types of cancers. Specifically, MIR31HG was found elevated expressed in breast cancer [13], cervical cancer [28], chordoma [16], osteosarcoma [15], lung cancer [2, 23, 41], OSCC [4], PDAC [7], VSCC [29] and LSCC [36, 42, 43]. Nevertheless, the expression levels of MIR31HG was reported down-regulated in triple-negative breast cancer (TNBC) cell lines of basal subtype [27], bladder cancer [26], gastric cancer [25], CRC [32, 33], and HCC [6]. Besides, abnormal expression of MIR31HG was deemed as potential biomarker to reflect the clinicopathological characteristics and prognostic outcomes of cancer patients [28]. For instance, increased MIR31HG expression was found both in LUAD/NSCLC tissues when compared with counterparts, and positively associated with unfavorable TNM stage, metastasis, histological differentiated degree, and forecasted shorter OS [2, 23, 41]. Notably, MIR31HG expression was even higher in gefitinib-resistant NSCLC cells, and knockdown of MIR31HG could promote cell apoptosis and cell cycle arrest, and subsequently induce gefitinib sensitivity [20]. More recently, He J et al. further verified that MIR31HG was also remarkably increased in gefitinib-resistant NSCLC patients, and MIR31HG overexpression contributed to reduced sensitivity of NSCLC cell to gefitinib in vitro [44]. By contrast, Yan S et al. reported that overexpression of MIR31HG significantly inhibited HCC proliferation and metastasis in vitro and impeded tumorigenesis in vivo [6]. In CRC, the MIR31HG expression was negatively correlated with unfavorable prognosis, as indicated by advanced pathologic stage, and lager tumor size [19]. Consistently, Li Y et al. found that high expression of MIR31HG predict high OS and DFS in CRC patients treated with oxaliplatin [32].

Given the prognostic significance of MIR31HG in cancers, in the current study, we firstly explored the association between MIR31HG level and clinical outcomes by performing a meta-analysis containing seventeen literatures with 2573 patients. The pooled results of subgroup analysis as per the tumor types demonstrated that high MIR31HG expression predicted unfavorable OS in patients with lung cancer and other cancers, and poor RFS in all selected studies, respectively. On the contrary, overexpression of MIR31HG was closely associated with favorable OS and DFS in gastrointestinal cancer, indicating a tissue-specific predictive prognosis of MIR31HG in multiple human cancers. Besides, the relationship between expression of MIR31HG and other parameters were analyzed. Consistent with the predictive value of MIR31HG in OS, high level of MIR31HG was also significantly correlated with advanced clinical stage and LNM potential in lung cancer. Previously, the remarkable associations between MIR31HG expressions with LNM risk or tumor stage in gastrointestinal cancer were not noted. However, we found that the study performed by Sun et al. may introduce bias to this meta-analysis, as shown in sensitivity analysis. Therefore, in order to increase the stability of the results, we excluded this study and found that elevated expression of MIR31HG were negatively associated with clinical stage and LNM risk in gastrointestinal cancer. However, no correlation between MIR31HG expression and other clinical characteristics, such as DM and tumor size, were noted. It is worth mentioning that since the studies with regard to the relationship between expression of MIR31HG and other clinical parameters are limited, these results could be biased and therefore needs further confirmation in future studies. Beside, in order to increase the credibility of the results, we performed cross-validation of the results by using TCGA dataset. The results showed the elevated MIR31HG was significantly correlated with poor OS rather than DFS in lung cancer. However, it should be noted that the enrolled studies reporting MIR31HG expression in lung cancers consisted of LUAD and NSCLC, while the cancer types in TCGA were LUAD and LUSC, which may lead to potential difference in predictive value of MIR31HG on clinical outcomes between this meta-analysis and TCGA dataset. Moreover, the limited sample size in TCGA may cause possible bias to the results.

Mechanistically, lncRNA could execute versatile functions in diseases through alternative splicing, epigenetic modulation, chromatin modification, scaffolding/decoy function or acting as molecular sponge [45]. To begin with, given the critical role of MIR31HG in hypoxia-associated malignant progression, MIR31HG was observed to promote tumorigenesis by serving as a HIF-1α co-activator and regulating HIF-1 transcriptional network [4, 42]. For instance, in LSCC, MIR31HG could facilitate cell proliferation, cell cycle progression and suppress apoptosis via HIF-1α and p21 [36]. In OSCC, MIR31HG knockdown impaired the HIF-1α transactivation, sphere-forming ability, metabolic shift and metastatic cascade both in vitro and in vivo [4]. Besides, lncRNA could involve in the competing endogenous RNA (ceRNA) regulatory work to function as endogenous miRNA sponge (Table 3) [6]. As the host gene of miR-31, MIR31HG was firstly identified to co-express or modulate the expression of miR-31 in certain cancers [44]. For example, MIR31HG could enhance proliferation, migration and invasion by up-regulating EZH2/miR-31 and then indirectly activating the oncogene RNF144B in chordoma [16]. Yang et al. demonstrated that elevated MIR31HG could inhibit miR-31 expression, and increase RhoA in LSCC, and via which promote cell growth, cell cycle and invasion [43]. In addition to miR-31, other miRNAs were also identified to interact with MIR31HG in carcinogenesis. It was reported that MIR31HG could directly sponge to tumor suppressor- miR-361, rather than miR-31, and in turn regulate cell proliferation, cell cycle arrest, and apoptosis via targeting VEGF, FOXM1 and Twist in osteosarcoma [15]. In NSCLC, MIR31HG behaved as an oncogene by inhibiting miR-214 expression, thereby facilitating cancer cell migration and invasion [41]. In HCC cancer, MIR31HG could competitively bind miR-575, and positively regulate ST7L to impair cell proliferation and metastasis [6]. Moreover, MIR31HG could regulate tumorigenesis by targeting a broad spectrum of target genes or pivotal signaling pathways (Table 3). In gastric cancer, MIR31HG may suppress cell proliferation and hinder tumorigenesis partly by regulation of E2F1 and p21 expression [25]. In NSCLC, MIR31HG was found to enhance Wnt/β-catenin pathway, and induce epithelial-mesenchymal transition (EMT) phenotype [2]. Jin et al. identified that MIR31HG was induced by nuclear translocation of NF-κB, and in turn directly bind to IκBα and participated in NF-κB activation, revealing an interaction between MIR31HG and NF-κB in osteogenic differentiation [46]. Of note, MIR31HG could modulate gefitinib resistance partly through EGFR/PI3K/Akt [20, 44] or RAF-MEK-ERK pathways [44]. Furthermore, Zheng S and colleagues discovered that down-regulation of MIR31HG inhibited NSCLC cell proliferation, invasion and EMT phenotype via up-regulating E-cadherin expression, inhibiting Wnt/β-catenin cascade, and down-regulating expression of Twist1 and vimentin [2].

**Table 3:** Expression pattern of MIR31HG in cancers and its interaction with miRNAs and target genes

Tumor type	Expression	Signaling pathways	MiRNAs and target genes	Biological functions	References
Osteosarcoma	↑	/	miR-361, VEGF, FOXM1, Twist	Cell proliferation, migration, apoptosis, G1/G arrest, EMT	[14]
Chordoma	↑	/	miR-31, EZH2, RNF144B	Cell proliferation, migration, invasion, EMT	[15]
OSCC	↑	/	HIF-1α, p300	Sphere-forming ability, metabolic shift and metastasis	[4]
HNSCC/LSCC	↑	/	HIF-1α, p21	Cell proliferation, cell cycle progression, apoptosis	[35]
LSCC	↑	/	miR-31, RhoA	Cell growth, cell cycle, invasion	[42]
Breast cancer	↑	/	/	Cell proliferation, apoptosis, migration, invasion	[12]
Cervical cancer	↑	/	/	Cell proliferation, apoptosis	[27]
VSCC	↑	/	/	/	[28]
NSCLC	↑	Wnt/β-catenin	GSK3β, Twist1, Vimentin, E-cadherin	Cell proliferation, invasion, EMT	[2]
NSCLC	↑	PI3K/Akt	EGFR	Cell proliferation, apoptosis, G0/G1 arrest, gefitinib resistance	[19]
NSCLC	↑	RAF/MEK/ERK, PI3K/Akt	miR-31, FIH-1, RASA1	Cell proliferation, clonogenic growth, gefitinib resistance	[43]
NSCLC	↑	/	miR-214	Cell migration, invasion	[40]
LUAD	↑	/	/	Cell proliferation, cell cycle arrest	[22]
PDAC	↑	/	miR-193b, CCND1, Mcl-1, NT5E, KRAS, uPA, ETS1	Cell growth, apoptosis, G1/S arrest, invasion	[6]
ESCC	↑	/	Furin, MMP1	Cell proliferation, migration, invasion	[29]
ESCC	↓	/	/	/	[30]
Gastric cancer	↑	/	p21, E-cadherin	Cell proliferation, migration	[33]
Gastric cancer	↓	/	E2F1	Cell proliferation	[24]
CRC	↑	/	miR-31-5p, MYC, TNF-α/NF-κB, TGF-β, IFN-α/γ	EMT	[16]
CRC	↓	/	/	Cell proliferation, apoptosis	[18]
HCC	↓	/	miR-575, ST7L	Cell proliferation, metastasis	[5]
Bladder cancer	↓	/	/	/	[25]

*Akt* protein kinase B, *CCND1* cyclin D1, *CRC*, colorectal cancer, *E2F1, E2F* transcription factor 1, *EGFR* epidermal growth factor receptor, *EMT* Epithelial-mesenchymal transition, *ERK* extracellular regulated protein kinases, *ESCC* esophageal squamous cell carcinoma, *ETS1, ETS* proto-oncogene 1, transcription factor, *EZH2* enhancer of zeste 2 polycomb repressive complex 2 subunit, *FIH-1* hypoxia inducible factor 1 subunit alpha inhibitor, *FOXM1* forkhead box M1, *GSK-3β* glycogen synthase kinase-3β, *HCC* hepatocellular carcinoma, *HIF-1α* hypoxia inducible factor 1 subunit alpha, *HNSCC* head and neck squamous cell carcinoma, *IFN-α/γ* interferon-α/γ, *LSCC* laryngeal squamous cell cancer, *LUAD* lung adenocarcinoma, *MMP1* matrix metallopeptidase 1, *NSCLC* Non–small cell lung cancer, *NT5E* 5′-nucleotidase ecto, *OSCC* oral squamous cell carcinoma, *PDAC* pancreatic ductal adenocarcinoma, *PI3K* phosphoinositide 3-kinase, *RASA1* RAS p21 protein activator 1, *RhoA* ras homolog gene family, member A, *ST7L* suppression of tumorigenicity 7 like, *TGF-β* transforming growth factor-β, *TNF* tumor necrosis factor, *VEGF* vascular endothelial growth factor, *VSCC* vulvar squamous cell carcinoma

↑up-regulated, ↓down-regulated

Despite an effort to make a comprehensive analysis, limitations remained inevitably in this study. First, most of the studies were among Asian populations though we do not impose restrictions on region during literature retrieval, which may introduce possible geographical bias. However, since these results have been further confirmed by TCGA dataset [21], it could be more credible to generalize the data to other populations. Second, some HRs with corresponding 95% CIs were indirectly extracted from the Kaplan–Meier (K-M) survival curves, which may introduce possible bias. Third, the cut-off value of MIR31HG levels varied among the selected studies, which may contribute to potential limitation to the clinical use.

## Conclusion

Taken together, our study provided evidence that MIR31HG overexpression suggested remarkable association with poor prognosis in terms of OS, tumor stage, and LNM in lung cancer, but favorable prognosis in gastrointestinal cancer. Therefore, MIR31HG may serve as a novel promising prognostic biomarker in patients with multiple cancers. However, more rigorously designed prospective studies with large sample size and complete clinical information are still requested for further confirmation of the results.

## Supplementary information

**Additional file 1: Figure S1.** Forest plots describing subgroup analysis of HRs for association between MIR31HG expression and OS based on follow-up months (**a**) and expression pattern of MIR31HG in cancerous tissues and matched normal samples in various cancers (**b**, **c**). *CI* confidence interval, *HR* hazard ratio, *OS* overall survival

## Data Availability

The datasets used and/or analyzed during the current study are available from the corresponding author on reasonable request.

## Abbreviations

Akt: Protein kinase B
BCAR4: Breast cancer anti-estrogen resistance 4
BLCA: Bladder urothelial carcinoma
BRCA: Breast invasive carcinoma
CCND1: Cyclin D1
ceRNA: Competing endogenous RNA
CESC: Cervical squamous cell carcinoma and endocervical adenocarcinoma
CI: Confidence interval
COAD: Colon adenocarcinoma
CRC: Colorectal cancer
DFS: Disease-free survival
DM: Distant metastasis
E2F1: E2F transcription factor 1
EGFR: Epidermal growth factor receptor
EMT: Epithelial-mesenchymal transition
ERK: Extracellular regulated protein kinases
ESCA: Esophageal carcinoma
ESCC: Esophageal squamous cell carcinoma
ETS1: ETS proto-oncogene 1, transcription factor
EZH2: Enhancer of zeste 2 polycomb repressive complex 2 subunit
FIGO: FOXM1: forkhead box M1
GEPIA: Gene Expression Profiling Interactive Analysis
GSK-3β: Glycogen synthase kinase-3β
HCC: Hepatocellular carcinoma
HIF-1α: Hypoxia inducible factor 1 subunit alpha
HNSCC: Head and neck squamous cell carcinoma
HR: Hazard ratio
IFN-α/γ: Interferon-α/γ
K-M: Kaplan-Meier
lncRNA: Long noncoding RNA
LncHIFCAR: Long noncoding HIF-1α co-activating RNA
LNM: Lymph node metastasis
LSCC: Laryngeal squamous cell cancer
LUAD: Lung adenocarcinoma
LUSC: Lung squamous cell carcinoma
MIR31HG: MicroRNA-31 host gene
MMP1: Matrix metallopeptidase 1
NOS: Newcastle–Ottawa Scale
NSCLC: Non‑small cell lung cancer
NT5E: 5′-Nucleotidase ecto
OS: Overall survival
OSCC: Oral squamous cell carcinoma
PAAD: Pancreatic adenocarcinoma
PDAC: Pancreatic ductal adenocarcinoma
PFS: Progression-free survival
PI3K: Phosphoinositide 3-kinase
qRT-PCR: Real-time quantitative reverse transcription polymerase chain reaction
READ: Rectum adenocarcinoma
RFS: Relapse-free survival
RhoA: Ras homolog gene family, member A
SARC: Sarcoma
SNHG16: Small nucleolar RNA host gene 16
ST7L: Suppression of tumorigenicity 7 like
STAD: Stomach adenocarcinoma
TCGA: The Cancer Genome Atlas
TGF-β: Transforming growth factor-β
TTN-AS1: TTN antisense RNA 1
TNF: Tumor necrosis factor
VEGF: Vascular endothelial growth factor
VSCC: Vulvar squamous cell carcinoma
