# Supplementary figures and images for: The predictive value of lncRNA MIR31HG expression on clinical outcomes in patients with solid malignant tumors
Source: Cancer Cell Int. 2020 Apr 7;20:115. doi: 10.1186/s12935-020-01194-y (PMC7137300; doi:10.1186/s12935-020-01194-y)

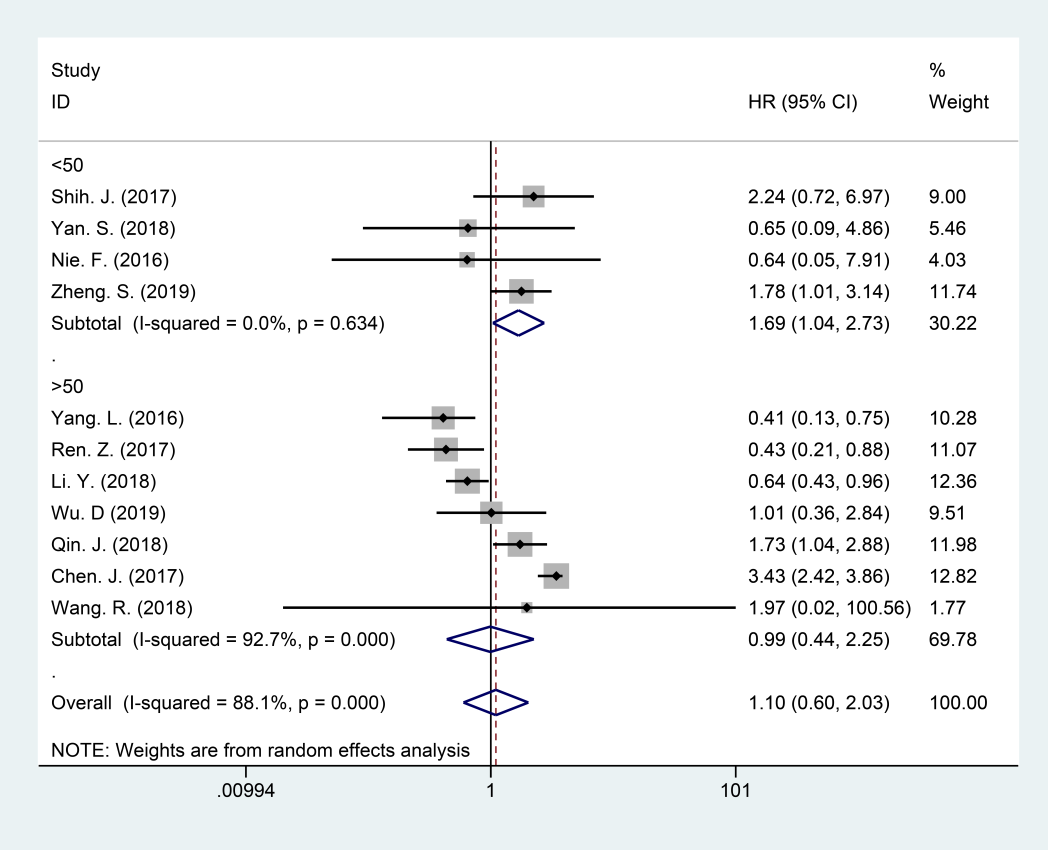


**A**

**B**


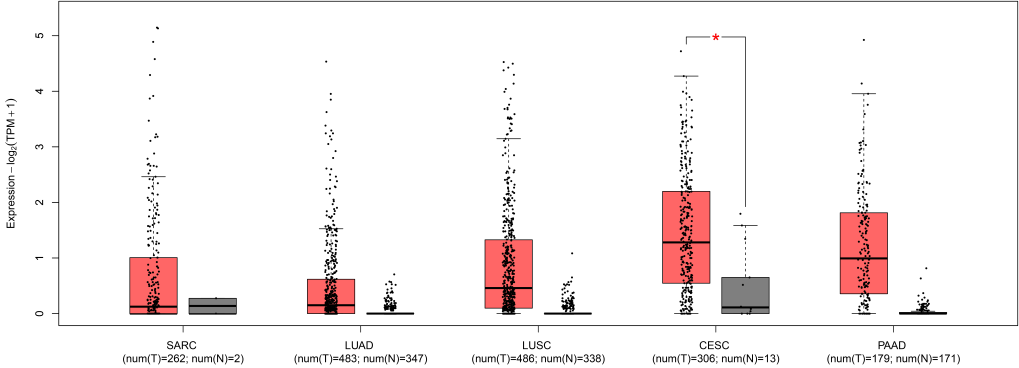


**C**


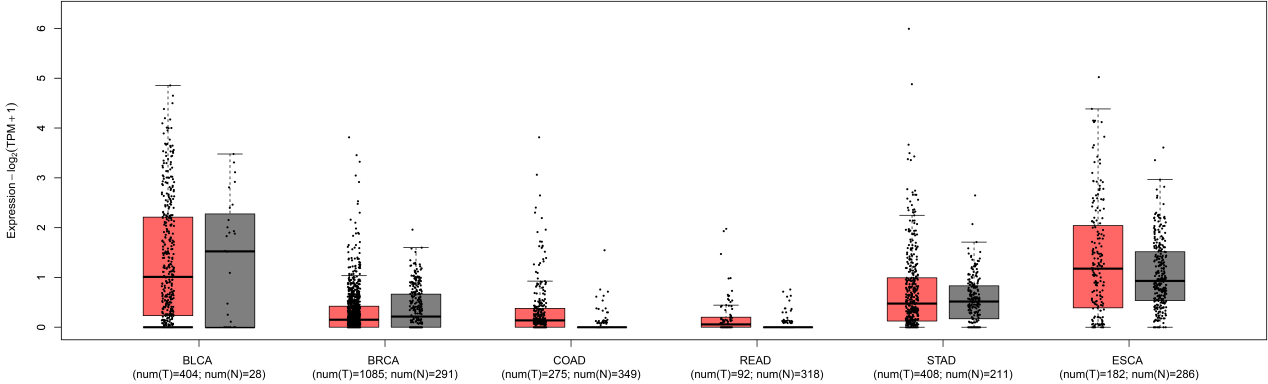


Figure S1

Supplement: Supplementary file 1 — Additional file 1: Figure S1. Forest plots describing subgroup analysis of HRs for association between MIR31HG expression and OS based on follow-up months (a) and expression pattern of MIR31HG in cancerous tissues and matched normal samples in various cancers (b, c). CI confidence interval, HR hazard ratio, OS overall survival [file 12935_2020_1194_MOESM1_ESM.docx]
